# Medical College of Alabama

**Published:** 1859-09

**Authors:** 


THE MEDICAL COLLEGE OF ALABAMA.—In our May number we announced that a new school was about being organized at Mobile, and since that time we have received the circular of the institution, from which we learn that the Chairs have been filled by the appointment of the following gentlemen: Dr. J. C. Nott, Surgery; Dr. J. F. Heustis, Anatomy; Dr. W. H. Anderson, Physiology and Pathology; Dr. G. A. Ketchum, Practice of Medicine; Dr. F. A. Ross, Materia Medica and Therapeutics; Dr. F. E. Gordon, Obstetrics and Diseases of Women and Children; Dr. W. J. Taylor, Chemistry; and Dr. G. Owen, Demonstrator of Anatomy. But five American works are recommended as text-books.

